# Supplementary material for: A Qualitative Analysis of a Caregivers’ Experience of Complementary Feeding in a Population of Native Hawaiian, Other Pacific Islander and Filipino Infants: The Timing of the Introduction of Complementary Foods, and the Role of Transgenerational Experience
Source: Nutrients. 2022 Aug 10;14(16):3268. doi: 10.3390/nu14163268 (PMC9412982; doi:10.3390/nu14163268)
Supplement: Supplementary file 1 [file nutrients-14-03268-s001.zip › nutrients-1836048-supplementary.pdf]

Table S1: Interview script of leading and follow-up questions used in in-depth interviews with Native Hawaiian and Other Pacific Islander, and Filipino caregivers, *n* = 29.[a]

Interview Questions
1. Are you exclusively breastfeeding or not exclusively breastfeeding?
2. Tell me about your childhood.<br>a. Where did you grow up?<br>b. Who raised you?<br>c. What were some cherished memories from your childhood?<br>d. Tell me about what you ate growing up?
3. What does a healthy baby look like?<br>a. How do you know if a baby is sick?<br>b. What foods do you think are considered healthy for babies?
4. How did you learn about what to feed your baby?<br>a. Who are your *kumu* (teachers)?<br>b. What are your *kumu's* skills?<br>c. Where are they from?
5. What do you feed your baby?<br>a. How is your baby's food prepared? Mashed? Pureed? Pre-chewed?<br>b. Who prepares your baby's food?<br>c. Where do you get your baby's food?
6. Who do you go to for advice about feeding your baby?<br>a. Do you go to a doctor? Nurse? Parent expert? Family?<br>b. If someone gave you advice would you follow it?<br>Were roles determined by gender or age? Example: *Kūpuna* are always older than the baby.
7. When did you feed your baby something other than milk? Milk being breastmilk, formula, condensed milk, cow's milk or any other kind of milk.<br>a. Was this different from what you were told to do?
8. What are your earliest recollections about how baby(ies) were fed in your family?<br>a. What were the roles of other family members in feeding the baby?<br>b. Were roles determined by age or gender?
9. What ways of feeding babies have remained among your family?<br>a. In your family, how has the feeding of babies changed over time from your generation to the next?
10. Through your life experiences how has your perception of feeding babies changed?<br>a. What in our community supports parents in serving traditional Hawaiian foods to their babies?<br>b. What makes it difficult for parents who want to serve traditional Hawaiian foods to their babies?

[a] *Shaded rows indicate the questions being analyzed in this study*
